# Simultaneous determination of colistin sulfate and tigecycline in human plasma by liquid chromatography-tandem mass spectrometry method

**DOI:** 10.1186/s13065-023-01109-8

**Published:** 2024-01-13

**Authors:** Ying-Chao Ma, Xi-Kun Wu, Xiu-Ling Yang, Zhi-Qing Zhang

**Affiliations:** Department of Pharmacy, Second Hospital of Hebei Medical University, No. 215 of Heping West Road,Xinhua District, Shijiazhuang, 050000 China

**Keywords:** Colistin sulfate, Tigecycline, Liquid chromatography–tandem mass spectrometry, Plasma drug concentration, Therapeutic medication monitoring

## Abstract

**Objective:**

To establish a high-performance liquid chromatography–tandem mass spectrometry method (HPLC–MS/MS) to simultaneously determine colistin sulfate and tigecycline in human plasma.

**Methods:**

Polymyxin B_1_ internal standard (20 µL) was added into 200 µL of plasma sample. The samples were treated with methanol-5% trichloroacetic acid (50:50, V/V) solution, and the protein precipitation method was adopted for post-injection analysis. The chromatographic column was a Dikma C18 (4.6 mm × 150 mm, 5 μm). For the mobile phase, 0.1% formic acid in aqueous solution was used for phase A, 0.1% formic acid in acetonitrile solution for phase B, and gradient elution was also applied. The flow rate was 0.8 mL/min, the column temperature was 40 °C, and the injection volume was 10 µL; Electrospray ionization and multiple reaction ion monitoring were adopted and scanned by the HPLC–MS/MS positive ion mode.

**Results:**

The endogenous impurities in the plasma had no interference in the determination of the analytes. There existed a good linear relationship of colistin sulfate within the range of 0.1–10 µg/mL (R^2^ = 0.9986), with the lower limit of quantification (LLOQ) of 0.1 µg/mL. There existed a good linear relationship of tigecycline within the range of 0.05–5 µg/ mL (R^2^ = 0.9987), with the LLOQ of 0.05 µg/mL. The intra- and inter-day relative standard deviations of colistin sulfate and tigecycline were both less than 15%, and the accuracy was between 88.21% and 108.24%. The extraction had good stability, the extraction recovery rate was 87.75–91.22%, and the matrix effect was 99.40–105.26%.

**Conclusion:**

This study successfully established a method for simultaneously detecting colistin sulfate and tigecycline plasma concentrations. The method was simple, rapid, and highly sensitive and could be applied for therapeutic medication monitoring.

## Introduction

Bacterial drug resistance has become a major challenge in the global public health sector. The emergence of multidrug resistance, extensive drug resistance, and pan-resistant bacteria have seriously threatened human health [1]. At present, among many drug-resistant bacteria, the most important ones are carbapenem-resistant Enterobacteriaceae and *Acinetobacter baumannii* [2], which cause high mortality and few therapeutic medications. Antibiotic monotherapy for these bacteria is often unsatisfactory, and combination therapy is required. The combination of polymyxin and tigecycline is often adopted clinically for critically ill patients with pan-resistant bacterial infections. Colistin sulfate (also known as polymyxin E) is a cationic polypeptide antibiotic containing multiple components. It was isolated from *E. coli* in 1950. The two main components of colistin sulfate are polymyxin E_1_ and polymyxin E_2_, which account for over 85% of the total content of colistin sulfate [3], which determines its blood concentration and clinical antibacterial activity [4]. Therapeutic drug monitoring (TDM) measures the combined concentration of these two components as the blood concentration of colistin sulfate. Tigecycline is a glycylcycline antibacterial drug that binds to the bacterial ribosomal subunit 30 S, blocking protein synthesis, and thus exhibiting antibacterial effects [5]. It is mainly used clinically to treat severe infections caused by gram-positive and gram-negative bacteria (excluding *Pseudomonas aeruginosa*), especially those caused by multidrug-resistant bacteria and pan-resistant bacteria. The combined use of these two drugs is considered the last line of defense for the treatment of these multidrug-resistant bacterial infections in clinical practice [6]. Currently, there are two types of polymyxin E available for clinical use: polymyxin sulfate and colistin methanesulfonate (CMS). The two have different structures, with CMS being the inactive prodrug of polymyxin E, requiring in vivo metabolism to exert its antibacterial effect. In contrast to CMS, polymyxin sulfate acts directly in the body, showing superior in vivo bactericidal activity compared to CMS. Furthermore, only 20–25% of the CMS is converted to polymyxin in patients with normal renal function, and significant interindividual variability exists. Consequently, in critically ill patients, it takes a longer time and larger doses to achieve effective blood drug concentrations, potentially delaying the treatment of patients with infections. Moreover, CMS is primarily cleared through the kidney, leading to a greater renal burden, while polymyxin sulfate is mainly cleared through other routes. With the increasing occurrence of carbapenem-resistant organisms in clinical settings, the use of polymyxin has become more widespread, especially given its superior antibacterial activity and lower renal burden. Due to the early launch of colistin sulfate and lack of modern drug development procedures, it has limited pharmacokinetic, pharmacological, and toxicological data. Therefore, the therapeutic window of colistin sulfate is narrow, and the incidence of adverse reactions is high [7, 8]. There are significant individual differences in clinical practice, requiring blood concentration monitoring. With the increasing use of tigecycline, it has been found to have significant individual differences in blood concentration, and even an increased risk of death after use [9]. However, large individual differences exist during the clinical application of the two drugs, especially in patients with severe infection. Due to this pathophysiological condition, plasma drug concentration monitoring is necessary to achieve the correct individualized dosing [10, 11]. Currently, there are methods for determining the plasma concentration of polymyxin, including capillary electrophoresis, high-performance liquid chromatography (HPLC) [12, 13], and HPLC–MS/MS positive ion mode [14]; and for determining the plasma concentration of tigecycline, including HPLC, reversed-phase-HPLC, as well as HPLC–MS/MS positive ion mode. In addition, there are few methods available for simultaneous measurement of both drugs, and only Barco et al. reported a method for simultaneous measurement of 14 antibiotics, including colistin sulfate and tigecycline [15]. However, in Barco’s method, two different protein precipitation extraction methods were used for polymyxin sulfate and tigecycline sample processing, and different internal standard substances were selected for different drugs. Thus, it is not a simultaneous determination method for polymyxin sulfate and tigecycline. Moreover, in Barco’s method, the quantification limits for polymyxin E1, polymyxin E2, and tigecycline are 0.3 mg/L, 0.5 mg/L, and 1 mg/L, respectively, which do not fully meet the clinical testing requirements. This study aims to establish an HPLC–MS/MS method to simultaneously determine the plasma concentrations of colistin sulfate and tigecycline in human plasma. It is a simple experiment to operate, with a short analysis time and high sensitivity, and it could lay a foundation for individualized medication in the future.

## Materials and methods

### Medications, instruments, and samples

The medications used were a colistin sulfate reference substance (content: 95.1%, batch number: 833,621, Dr. Ehrenstorfer Co., Ltd., Germany), tigecycline reference substance (content: 99.6%, batch number: 04919009, Lianyungang Runzhong Pharmaceutical Co., Ltd.), and polymyxin B_1_ (content: ≥95%, batch number: P037-01BL, TOKU-E, USA).

The instruments used were an LC-20 C high-performance liquid chromatograph (Shimadzu Co., Japan), API4000 mass spectrometer (Applied Biosystem Co., USA), electronic analytical balance (Sartorius, Germany), high-speed centrifuge (ABBOTT Co., USA), and a low-temperature refrigerator (Sanyo, Japan).

Plasma samples were collected from patients receiving simultaneous intravenous polymyxin and tigecycline treatment. Inclusion criteria: ① Patients receiving simultaneous intravenous polymyxin and tigecycline treatment; ② Age ≥ 18 years; ③ Patients who consent to the monitoring of polymyxin and tigecycline blood drug concentrations during treatment. Exclusion criteria: ① Pregnant or lactating women; ② Treatment duration < 3 days; ③ Local administration; ④ Blood dialysis treatment. Once the blood drug concentration reached a steady state, 3 ml of venous blood was collected before and 30 min after the seventh administration to measure the peak and trough concentrations. This study was conducted with approval from the Ethics Committee of Second Hospital of Hebei Medical University (2020-R551). This study was conducted in accordance with the declaration of Helsinki.

### Detection conditions

#### Chromatographic conditions

The chromatographic column was a Dikma C18 chromatographic column (4.6 mm×150 mm, 5 μm). For the mobile phase, phase A was the 0.1% formic acid in aqueous solution, and phase B was the 0.1% formic acid in acetonitrile solution. The flow rate was 0.8 mL/min, the column temperature was 40 °C, and gradient elution was adopted. The elution method was as follows: 0–1 min 5% phase B solution; 1–5 min 5–60% phase B solution; 5–6 min 60–95% phase B solution; 6–7 min 95% phase B solution; 7–9 min 95–5% phase B solution; 9–10 min 5% phase B solution.

#### Mass spectrometry conditions

**Fig. 1:**
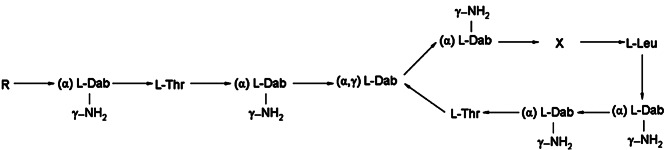
Chemical structure of polymyxin E_1,_ polymyxin E_2,_ and polymyxin B_1_. Dab = L-α,γ-diaminobutyric acid. α and γ indicate the respective-NH_2_ involved in the peptide linkage. Polymyxin B_1_: R = (+)-6-methyloctanoate, X = Phe; polymyxin E_1_: R = (+)-6-methyloctanoate, X = D-Leu; polymyxin E_2_: R = (+)-6-methylheptanoate, X = D-Leu

**Fig. 2:**
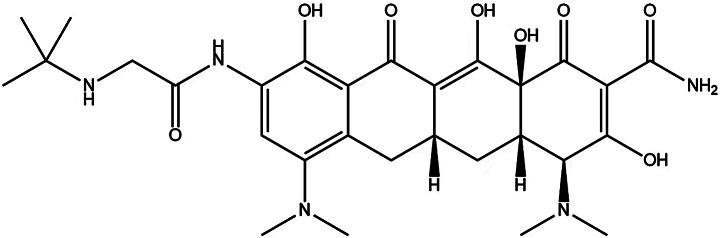
Chemical structure of tigecycline

**Fig. 3:**
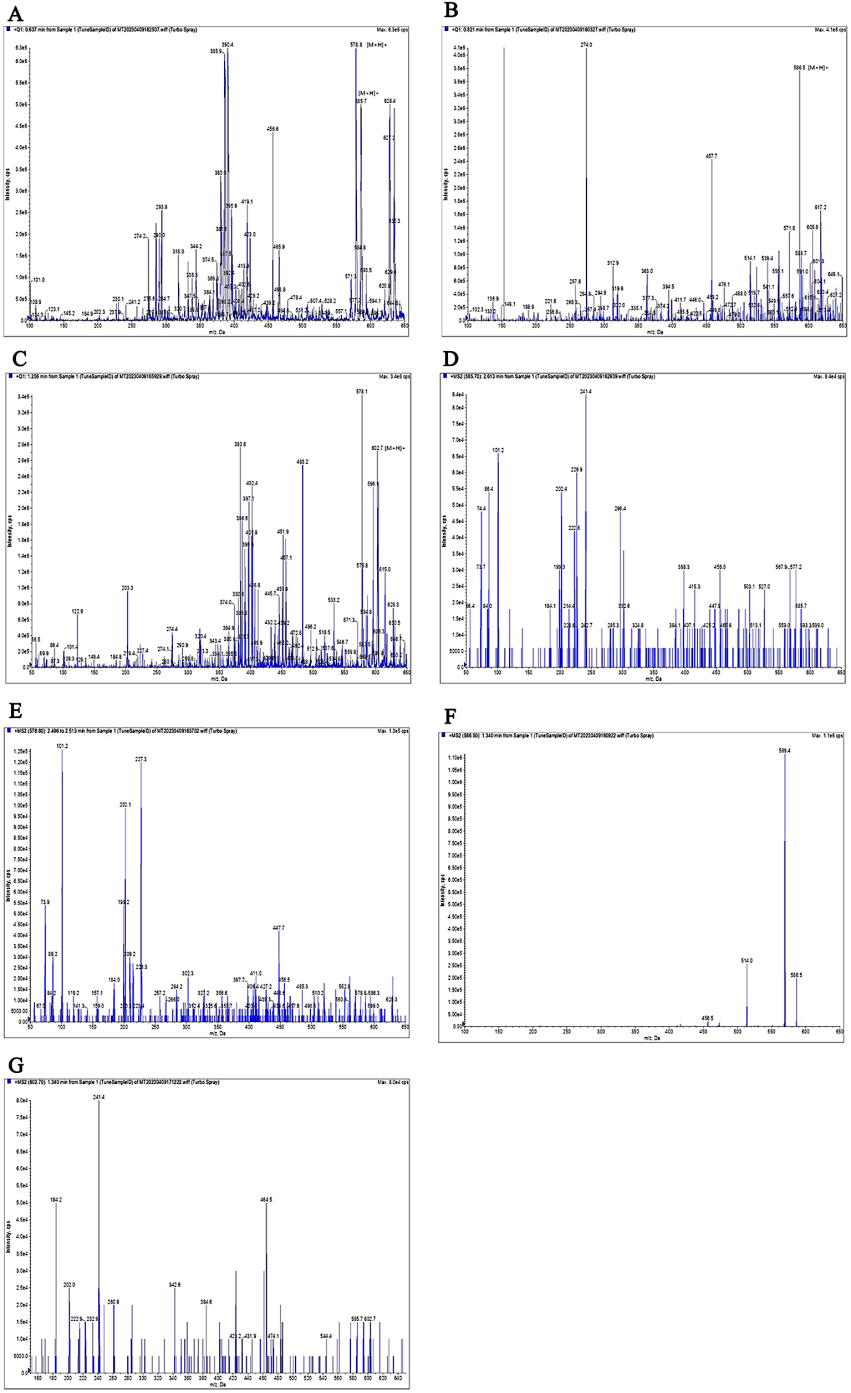
MS spectra of polymyxin E_1_, polymyxin E_2_, tigacycline and polymyxin B_1_. (**A**) precursor ion of Polymyxin E_1_ and polymyxin E_2_; (**B**) precursor ion of tigecycline; (**C**) precursor ion of polymyxin B_1_; (**D**) product ion of polymyxin E_1_; (**E**) product ion of Polymyxin E_2_; (**F**) product ion of tigecycline; (**G**) product ion of Polymyxin B_1_

Electrospray ionization (ESI), multiple reaction ion monitoring, and the HPLC–MS/MS positive ion mode were adopted. The voltage of the ESI was 5500 V with a temperature of 550 °C. The curtain air pressure was 10 psi, the atomizer pressure was 55 psi, the auxiliary air pressure was 55 psi, and the impact air pressure was 4 psi. The mass-to-charge ratios (m/z) from the quantitative analysis of polymyxin E_1_, polymyxin E_2_, tigecycline, and internal standard polymyxin B_1_ were 585.7→101.2, 578.8→101.2, 586.5→569.4 and 602.7→241.4, respectively, with de-clustering potentials of 61, 59, 110 and 68 V, respectively, and impact potentials of 47, 49, 30 and 33 V, respectively. The structural diagrams of polymyxin E_1_, polymyxin E_2_, tigecycline, and the internal standard are shown in Figs. 1 and 2. Their mass spectrometry (MS) spectra are shown in Fig. 3.

### Solution preparation

#### Reference solution

Twenty milligrams of colistin sulfate reference substance were precisely weighed, placed in a 10 mL volumetric flask, and diluted to volume with 20% methanol-aqueous solution (*v*:*v*) to prepare a stock solution of colistin sulfate with a mass concentration of 2 mg/mL. Ten mg of tigecycline reference substance was precisely weighed, placed in a 10 mL volumetric flask, and diluted to volume with pure water to prepare a tigecycline stock solution with a mass concentration of 1 mg/mL. The above solutions were stored in a − 80 °C refrigerator in preparation for further assay.

The colistin sulfate and tigecycline reference stock solutions were precisely aspirated, diluted, and mixed with 20% methanol-aqueous solution (*v*:*v*) to prepare the standard curve working solutions of a colistin sulfate mass with concentrations of 1, 2, 5, 10, 20, 50, and 100 µg/mL, and of tigecycline with concentrations of 0.5, 1, 2, 5, 10, 20, and 50 µg/mL, respectively. The quality control working solutions of colistin sulfate with concentrations of 1, 10, and 80 µg/mL and that of tigecycline with concentrations of 0.5, 5, and 40 µg/mL were prepared according to the above methods. The above working solutions were stored in a − 80 °C refrigerator for further assay.

#### Internal standard solution

Twenty milligrams of polymyxin B_1_ reference substance was precisely weighed, placed in a 10 mL volumetric flask, and diluted to volume with 20% methanol-aqueous solution (*v*:*v*) to prepare an internal standard stock solution with a mass concentration of 2 mg/mL and was stored in a − 80 °C refrigerator for further assay. A certain amount of stock solution was then diluted to a solution with a concentration of 40 µg/mL with 20% methanol-aqueous solution (*v*:*v*).

### Plasma sample processing

Two hundred microliters of the plasma sample were adopted, with 20 µL of the internal standard added, and it was vortexed for 20 s. Two hundred microliters of 5% trichloroacetic acid was added for acidification and vortexed for 10 s. Two hundred microliters of methanol was added for extraction, vortexed for 2 min, and centrifuged at 10,900 r/min for 5 min. Two hundred microliters of the supernatant were aspirated into the injection bottle, and 10 µL of the sample was injected for the HPLC–MS/MS analysis.

### Methodological investigation

#### Specificity

A total of 160 µL of blank plasma was taken, and 20 µL each of polymyxin working solution and tigecycline working solution were added. After vortex mixing, 200 mL of plasma samples from patients receiving simultaneous intravenous polymyxin and tigecycline treatment were taken and processed according to the “Plasma Sample Handling” procedure. Then, 200 µL of blank plasma was also taken and mixed with 20% methanol-water solution (*v:v*) 20 µL without internal standards. After adding 20 µL of internal standard working solution, it was used as a blank control for HPLC-MS/MS analysis, and the chromatogram was recorded.

#### Standard curve and limits of quantitation

One hundred eighty microliters of the blank plasma were adopted, with 20 µL of standard curve working solution and 20 µL of internal standard solution added, and it was vortexed for 20 s. Then, the standard curve plasma solutions of colistin sulfate with concentrations of 0.1, 0.2, 0.5, 1, 2, 5, and 10 µg/mL and those of tigecycline with concentrations of 0.05, 0.1, 0.2, 0.5, 1, 2, and 5 µg/mL were prepared according to the operations in the “plasma sample processing.” Three replicates were prepared for each concentration, and the HPLC–MS/MS analysis was conducted to determine the peak area. The concentration of the analyte was selected as the *x*-coordinate and the peak area ratio of the analyte to the internal standard as the *y*-coordinate, and the least squares method was adopted for weighted linear regression to obtain the standard curve. The peak area of colistin sulfate was calculated as the sum of the peak areas of polymyxin E_1_ and polymyxin E_2_. The limits of quantitation (LOQ) for the concentration analysis of colistin sulfate and tigecycline were determined with a signal-to-noise (S/N) ≥ 10.

#### Precision and accuracy

One hundred eighty microliters of the blank plasma was adopted, with 20 µL of quality control working solution and 20 µL of internal standard solution added, and it was vortexed for 20 s. Three quality control plasma samples were then prepared with low, medium, and high mass concentrations (the mass concentrations of colistin sulfate were 0.1, 1, and 8 µg/mL, respectively, and those of tigecycline were 0.05, 0.5, and 4 µg/mL, respectively), according to the operations in “plasma sample processing.” Five replicates of each concentration were prepared in parallel and conducted according to the operations in “plasma sample processing” for three consecutive days. The obtained peak area was introduced into the standard curve of that day to calculate the relative standard deviation (RSD) and accuracy of the intra-day and inter-day precision of the two analytes.

#### Extraction recovery rate and matrix effect

One hundred eighty microliters of the blank plasma was adopted, with 20 µL of the quality control working solution and 20 µL of the internal standard solution added, and it was vortexed for 20 s to prepare the plasma samples with low, medium, and high mass concentrations (among which the mass concentrations of colistin sulfate were 0.1, 1, and 8 µg/mL, respectively, and those of tigecycline were 0.05, 0.5, and 4 µg/mL, respectively). The plasma samples were then processed according to the operations in “plasma sample processing” and labeled as sample (A) In addition, blank plasma was processed according to the operations in “plasma sample processing” and then added with a series of quality control solutions to obtain plasma samples with low, medium, and high mass concentrations (among which the concentrations of colistin sulfate were 0.1, 1, and 8 µg/mL, respectively, and those of cyclocycline were 0.05, 0.5, and 4 µg/mL, respectively), which were labeled as sample (B) A standard mixed solution with the same concentration was prepared as sample (C) Five replicates of each concentration were prepared. The three groups of samples were analyzed by HPLC–MS/MS in the same way. The extraction recovery rate was calculated by the ratio of the peak area of sample A to that of sample B, and the ratio of the peak area of sample B to that of sample C was used to calculate the matrix effect.

#### Stability

Plasma samples with low, medium, and high mass concentrations were prepared (the mass concentrations of colistin sulfate were 0.1, 1, and 8 µg/mL, respectively, and those of tigecycline were 0.05, 0.5, and 4 µg/mL, respectively). Five replicates of each concentration were prepared and placed in a refrigerator at 4 °C for 12 h, at room temperature for 6 h, and in an automatic sampler at 15 ℃ for 24 h. The samples were, on three occasions, frozen at − 80 °C and thawed at room temperature, and then placed in a − 80 °C refrigerator for seven days. The concentrations and RSDs of colistin sulfate and tigecycline in the plasma samples were calculated based on the accompanying standard curve of the day to evaluate their stabilities.

## Results

### Methodological evaluation

#### Specificity

**Fig. 4:**
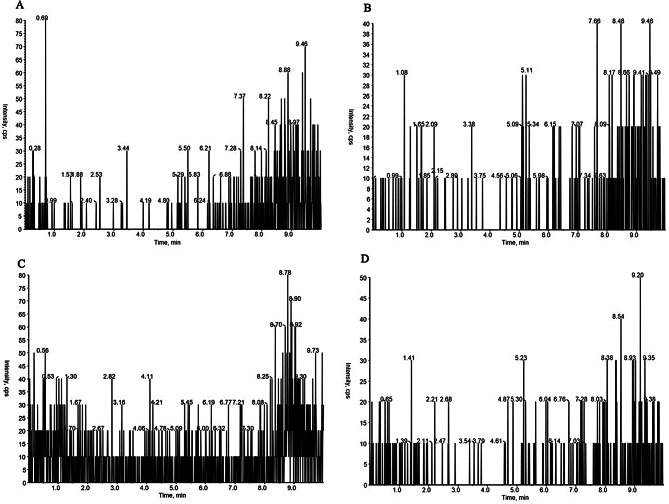
MRM chromatograms for blank plasma (**A** polymyxin E_1_; **B** polymyxin E_2_; **C** tigecycline; **D** polymyxin B_1_)

**Fig. 5:**
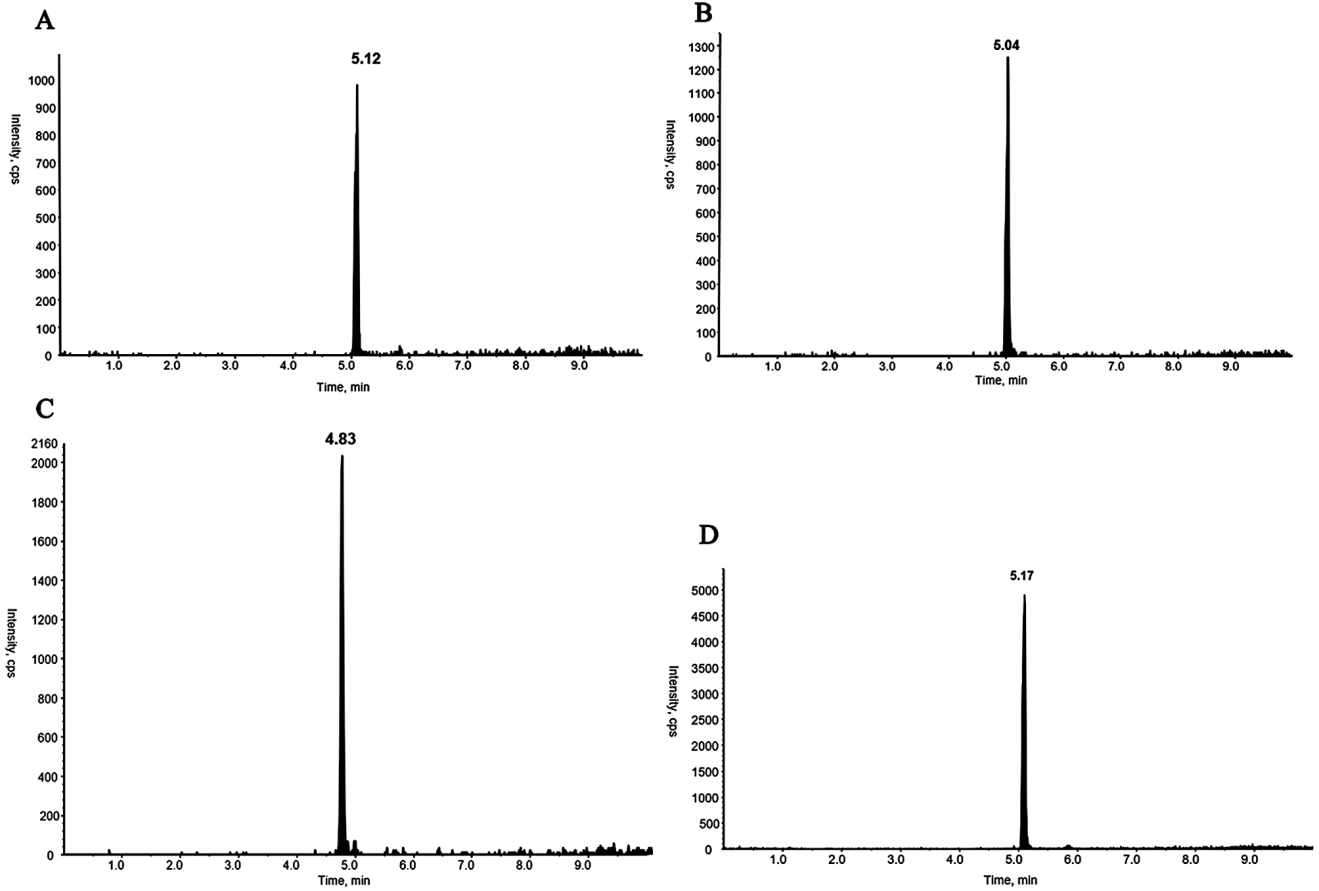
MRM chromatograms for blank plasma with LOQ levels of colistin sulfate, tigecycline, and internal standard (**A** polymyxin E_1_; **B** polymyxin E_2_; **C**, tigecycline; **D**, polymyxin B_1_)

**Fig. 6:**
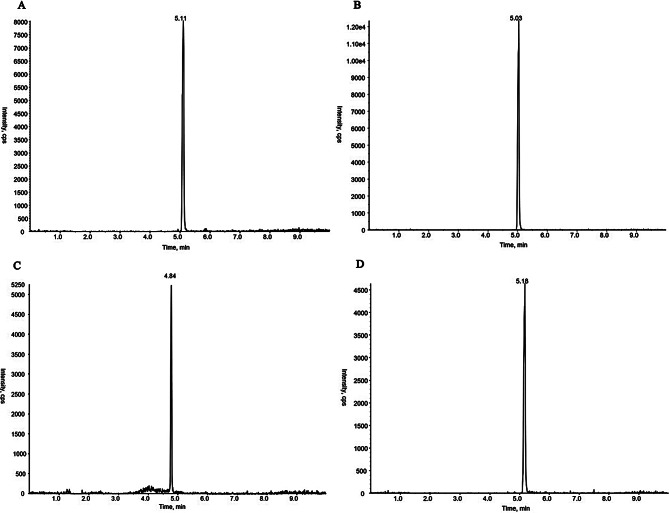
MRM chromatograms for blank plasma with colistin sulfate (2 µg/mL), tigecycline (0.2 µg/mL), and internal standard (40 µg/mL) (**A** polymyxin E_1_; **B** polymyxin E_2_; **C** tigecycline; **D** polymyxin B_1_)

**Fig. 7:**
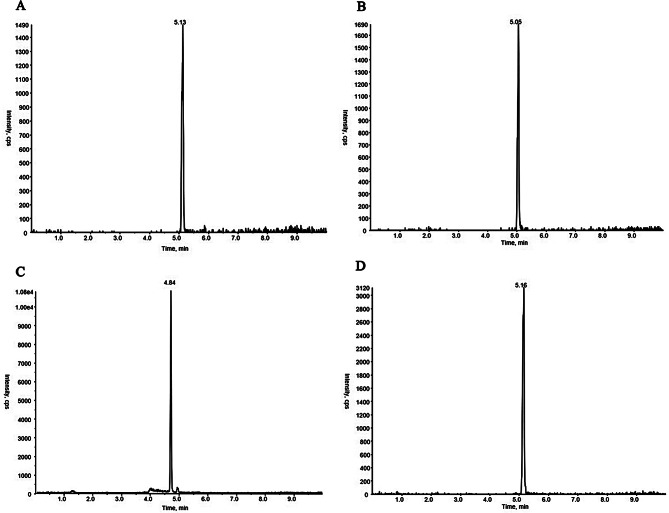
MRM chromatograms for clinical plasma with the internal standard (**A** polymyxin E_1_; **B** polymyxin E_2_; **C** tigecycline; **D** polymyxin B_1_)

Under the chromatographic conditions in this experiment, the total running time of HPLC–MS/MS analysis was 10 min, and the retention times of the four substances, including polymyxin E_1_, polymyxin E_2_, tigecycline, and internal standard, were 5.11 min, 5.03 min, 4.84 min, and 5.18 min, respectively. The endogenous impurities in the plasma samples did not interfere with the analytes and internal standards and could be adopted for quantitative analysis. The chromatograms are shown in Figs. 4, 5, 6 and 7.

#### Standard curve and limits of quantitation

A good linear relationship existed, with the concentration of colistin sulfate and tigecycline in the plasma within the range of 0.1–10 µg/mL and 0.05–5 µg/mL, respectively. The standard curve equations were *y* = 0.814*x* + 0.056 (R^2^ = 0.9986), *y* = 5.012*x* − 0.052 (R^2^ = 0.9987), and the LLOQs were 0.1 µg/mL and 0.05 µg/mL, respectively.

#### Precision and accuracy

The RSD of the intra-day and inter-day precision of colistin sulfate and tigecycline were both less than 15%, with the accuracy being within the range of 88.21–108.24%. The results are demonstrated in Table 1.

**Table 1:** The intra- and inter-day relative standard deviation and accuracy (n = 5)

Analyte	Quality control concentration (µg/mL)	Intra-day relative standard deviation			Inter-day relative standard deviation		
		Measured value (µg/mL)	RSD (%)	Accuracy (%)	Measured value (µg/mL)	RSD (%)	Accuracy (%)
Colistin sulfate	0.1	0.09 ± 0.00	4.10	88.21	0.10 ± 0.01	8.45	97.77
	1	1.08 ± 0.03	2.73	107.98	1.01 ± 0.07	6.60	101.12
	8	7.89 ± 0.28	3.58	98.65	7.94 ± 0.27	3.37	99.30
Tigecycline	0.05	0.05 ± 0.00	3.19	108.24	0.05 ± 0.00	7.16	104.15
	0.5	0.47 ± 0.02	3.24	93.97	0.48 ± 0.03	5.90	95.99
	4	4.20 ± 0.39	9.32	104.97	4.13 ± 0.27	6.56	103.32

#### Extraction recovery rate and matrix effect

The extraction recovery rates of colistin sulfate and tigecycline were 87.75–91.22%, and the matrix effect was 99.40–105.26%. The RSD was all less than 15%. The results showed that the analytes had a high recovery rate and were not affected by the matrix effect. The details are illustrated in Table 2.

**Table 2:** The extraction recovery rate and matrix effect (n = 5)

Analyte	Quality control concentration (µg/mL)	Extraction recovery rate (%)	RSD (%)	Matrix effect (%)	RSD (%)
Colistin sulfate	0.1	87.75 ± 7.74	8.82	99.40 ± 6.96	7.01
	1	88.61 ± 4.50	5.07	101.24 ± 2.64	2.60
	8	91.22 ± 2.62	2.88	102.53 ± 1.60	1.56
Tigecycline	0.05	88.89 ± 2.38	2.68	102.20 ± 5.37	5.25
	0.5	90.76 ± 4.56	5.02	102.77 ± 11.46	11.16
	4	90.05 ± 4.65	5.17	105.26 ± 2.37	2.25

#### Stability

The RSD of the concentration of colistin sulfate was 0.75–9.14%, and that of the concentration of tigecycline was 0.72–11.76%. The results showed that the analytes had good stability under the above conditions. The results are shown in Table 3.

**Table 3:** The stability of two analytes under different conditions (n = 5)

Analyte	Quality control concentration (µg/mL)	At 4℃ for 12 h		At room temperature for 6 h		Automatic sampler 15℃ for 24 h		Freeze-thaw for 3 cycles		Stored at -80℃ for 7 days	
		Measured value (µg/mL)	RSD (%)	Measured value (µg/mL)	RSD (%)	Measured value (µg/mL)	RSD (%)	Measured value (µg/mL)	RSD (%)	Measured value (µg/mL)	RSD (%)
Colistin sulfate	0.1	0.09 ± 0.01	8.21	0.10 ± 0.00	4.63	0.10 ± 0.00	4.47	0.09 ± 0.00	3.93	0.10 ± 0.00	4.15
	1	0.93 ± 0.04	4.85	1.00 ± 0.05	5.11	0.93 ± 0.01	0.75	1.04 ± 0.04	3.93	0.98 ± 0.09	9.14
	8	8.23 ± 0.61	7.39	8.26 ± 0.30	3.64	8.17 ± 0.19	2.38	8.70 ± 0.54	6.16	8.30 ± 0.32	3.86
Tigecycline	0.05	0.05 ± 0.00	3.95	0.05 ± 0.00	5.09	0.05 ± 0.00	1.64	0.05 ± 0.00	9.09	0.05 ± 0.00	2.55
	0.5	0.46 ± 0.01	1.99	0.54 ± 0.03	4.91	0.49 ± 0.03	5.29	0.52 ± 0.04	7.47	0.46 ± 0.03	6.05
	4	3.93 ± 0.36	9.19	4.87 ± 0.07	1.88	4.00 ± 0.26	6.46	3.82 ± 0.45	11.76	4.30 ± 0.03	0.72

### Example of application

This study included a total of 12 adult patients receiving intravenous polymyxin, consisting of 7 males and 5 females, with an average age of (58.67 ± 16.44) years. Two patients were administered an initial dose of 100 mg and a maintenance dose of 50 mg q12h for polymyxin, while the remaining 10 patients did not receive a doubled initial dose. All patients received an initial dose of 100 mg and a maintenance dose of 50 mg q12h for tigecycline. Three milliliters of venous blood were collected before and after the seventh administration to measure the peak and trough concentrations of the drugs. It was revealed that the trough concentration of colistin sulfate ranged from 0.15 to 2.51 µg/mL, the peak concentration ranged from 0.89 to 4.56 µg/mL, and the trough concentration of tigecycline ranged from 0.25 to 0.81 µg/mL, the peak concentration ranged from 0.92 to 1.78 µg/mL. The results of this method were all within the linear range of the study. The results are shown in Table 4.

**Table 4:** The plasma drug concentration of colistin sulfate and tigecyclin

No.	Gender	BMI (kg/m^2^)	CrCL^a^ (ml/min)	ALB^b^ (g/L)	Colistin sulfate dosage of administration	Colistin sulfate		Tigecycline dosage of administration	Tigecycline	
						Trough concentration(µg/mL)	Peak concentration(µg/mL)		Trough concentration(µg/mL)	Peak concentration(µg/mL)
1	Male	27.04	107	33.8	500,000IU q12h	0.26	1.13	First dose 100 mg maintenance dose 50 mg q12h	0.79	1.72
2	Female	27.69	36	30.35	First dose 1 million IU maintenance dose 500,000 IU q12h	2.02	4.56	First dose 100 mg maintenance dose 50 mg q12h	0.81	1.68
3	Male	23.88	90	26.80	500,000 IU q12h	0.28	1.22	First dose 100 mg maintenance dose 50 mg q12h	0.77	1.67
4	Male	18.72	111	32.50	500,000 IU q12h	0.28	1.39	First dose 100 mg maintenance dose 50 mg q12h	0.69	1.47
5	Male	22.14	84	28.00	500,000 IU q12h	0.84	2.51	First dose 100 mg maintenance dose 50 mg q12h	0.58	1.17
6	Femaee	19.3	161	35.3	500,000 IU q12h	0.15	0.89	First dose 100 mg maintenance dose 50 mg q12h	0.66	1.11
7	Male	24.16	59	36.72	500,000 IU q12h	0.78	2.33	First dose 100 mg maintenance dose 50 mg q12h	0.58	1.01
8	Male	27.44	98	33.30	500,000 IU q12h	0.39	1.93	First dose 100 mg maintenance dose 50 mg q12h	0.63	0.92
9	Female	20.91	106	32.71	500,000 IU q12h	0.44	2.27	First dose 100 mg maintenance dose 50 mg q12h	0.65	1.22
10	Female	21.95	129	31.89	500,000 IU q12h	0.37	2.03	First dose 100 mg maintenance dose 50 mg q12h	0.33	1.08
11	Male	22.51	27	27.10	First dose 1 million IU maintenance dose 500,000 IU q12h	2.51	4.27	First dose 100 mg maintenance dose 50 mg q12h	0.25	0.99
12	Female	23.71	170	32.50	500,000 IU q12h	0.17	1.09	First dose 100 mg maintenance dose 50 mg q12h	0.48	1.78

^a^Creatinine clearance on the day of blood collection

^b^White blood cell count on the day of blood collection

## Discussion

Currently, there is a lack of a internationally for simultaneously determining colistin sulfate and tigecycline in human plasma. Establishing the present method might provide a means of simultaneously determining the plasma drug concentrations for patients taking a combined medication of the two drugs, which could save costs and be convenient to operate without needing to switch the experimental methods. The present method saved the time of equilibrating the chromatographic column, preparing a new mobile phase, issuing a report, providing patients with plasma drug concentration data as soon as possible, and adjusting the individualized drug regimen in a timely manner. In addition, the present method had high accuracy, good reproducibility, and strong specificity, which could meet the requirements of pharmacokinetic investigations of colistin sulfate and tigecycline and lay a foundation for further pharmacokinetic/pharmacodynamic (PK/PD) investigations.

This study compared two pretreatment methods, solid-phase extraction and protein precipitation of samples. The solid-phase extraction method involves cumbersome operations with a run time of 20 min, leading to relatively time-consuming and material-consuming processes. Therefore, the protein precipitation method was ultimately selected for the processing of blood samples. Five reagents, including methanol, acetonitrile, trichloroacetic acid, trifluoroacetic acid, and perchloric acid, were compared for protein precipitation. The results indicated that all of the aforementioned reagents could be used for precipitation, but the recovery rate of polymyxin sulfate using acetonitrile was lower than that using methanol. Considering the widespread distribution of tigecycline in the body tissues and the need to avoid using strong acid precipitation that could corrode the chromatographic column, a low concentration of methanol-5% trichloroacetic acid (50:50, V/V) was ultimately chosen for protein precipitation to ensure a higher extraction recovery rate and simplify the plasma pretreatment process. This selection improved the analysis efficiency, making it suitable for clinical analysis with a large sample size. For the mobile phase, investigations were conducted using 0.05% formic acid aqueous solution-acetonitrile, 0.1% formic acid aqueous solution-acetonitrile, 0.2% formic acid aqueous solution-acetonitrile, and 0.1% formic acid aqueous solution-0.1% acetonitrile, followed by isocratic or gradient elution. Finally, considering the peak shape, elution time, and corrosive effects on the instrument, a 0.1% formic acid aqueous solution-0.1% acetonitrile solution was selected as the mobile phase for gradient elution. Under these conditions, the drug exhibited good resolution and peak shape.

The PK process of the drug in the body is often affected in patients with serious diseases accompanied by microcirculation disorders, hypoproteinemia, liver and kidney insufficiency, and other special pathophysiological states. Large fluctuations in plasma drug concentrations are commonly observed in these cases. TDM can aid in the optimization of administration dosage, individualized dosing, and anti-infective efficacy. Since colistin sulfate has not undergone modern drug development procedures, the current pharmacokinetic data remain unclear, and the plasma concentration is closely correlated with its antibacterial effect and nephrotoxicity [16]. In *Vitro* and animal studies revealed that the free drug area under the concentration-time curve to the minimum inhibitory concentration ratio (fAUC/MIC) is the PK/PD index that best correlates with the efficacy of colistin sulfate [17]. A study on the population PKs of colistin sulfate showed that when MIC ≤ 0.5 µg/mL, the recommended dosages of colistin sulfate were 500,000 IU q12h, 500,000 IU q8h, or 750,000 IU q12h, in which probability of target attainment (PTA) could reach > 90% in all schedules. However, when MIC = 1 µg/mL, for patients with creatinine clearance (CrCL) > 80 mL/min, there was a sub-optimal exposure risk at 500,000 IU q8h and 750,000 IU q12h; thus, a therapeutic schedule of 1,000,000 IU q12h was recommended. When MIC ≥ 2 µg/mL, all dosage schedules recommended in the instructions failed to achieve PTA ≥ 90% [18]. The blood drug concentration of colistin sulfate in 12 patients was detected using this method, the trough concentration range was 0.15 to 2.51 µg/mL, and the peak concentration range was 0.89 to 4.56 µg/mL. The large difference is due to the fact that patient 2 and patient 11 were given a double-dose of colistin sulfate as the first dose, compared to the recommended dose of 500,000 IU q12h. Additionally, patient 2, patient 2 and patient 11 had renal insufficiency, and colistin sulfate is primarily excreted through the kidneys, with 40% of the administered dose excreted in the urine within 8 h after administration. In patients with renal insufficiency, the drug tends to accumulate in the body, leading to high blood concentrations, hence the simultaneous effect of the double-dose and renal insufficiency. The results showed that there was a large individual difference in the pharmacokinetics of colistin sulfate, and the patient’s renal function affected drug excretion. The dosing regimen of 500,000 IU q12h specified in the instructions is insufficient, and a double dose administration scheme should be adopted to increase the blood drug concentration and improve clinical efficacy. In this study, all 12 patients received tigecycline at the recommended doses as per the instructions, with an initial dose of 100 mg and a maintenance dose of 50 mg q12h. The trough concentration range of tigecycline was 0.25 to 0.81 µg/mL, while the peak concentration ranged from 0.92 to 1.78 µg/mL, demonstrating significant interindividual differences. A study on the clinical efficacy of tigecycline in patients with severe infections showed that when the albumin(ALB) level < 26 g/L and the fAUC_0–24 h_/MIC > 0.9, the clinical antibacterial efficacy was reduced by nearly half of that in patients with an albumin level > 26 g/L [19]. The reason for this is that hypoalbuminemia can reduce the binding of albumin to tigecycline, leading to an increase in free drug concentration and an apparent increase in the volume of distribution. An increase in the free form of the drug can enhance renal clearance, further reducing drug concentration, thus affecting the therapeutic effect. Therefore, the changes in a patient’s albumin levels affect its therapeutic efficacy. Another PK/PD investigation on tigecycline in patients with severe diseases confirmed that for patients with intra-abdominal infections and community-acquired pneumonia, when MIC ≥ 1 µg/mL, the AUC/MIC compliance rate of patients treated with conventional tigecycline (with the first dose of 100 mg and a maintenance dose of 50 mg q12h) was significantly reduced, thus an increase in dosage was necessary [19, 20]. Moreover, body mass index (BMI) can also affect the volume of distribution of polymyxin and tigecycline in the body, serving as an important determinant for the specific dosage administered. It has been claimed that patients with a high BMI may require increased antibiotic dosages; however, this point is still controversial and requires further exploration.

This study had a relatively small sample size, with all patient BMIs falling within the normal range and all patient ALB values exceeding 26.00 g/L. Only three patients had renal insufficiency, indicating the need for a larger sample size and more comprehensive clinical data to obtain pharmacokinetic data and evaluate the relationship between blood drug concentration and clinical efficacy. The method established in this study for the simultaneous determination of polymyxin and tigecycline blood drug concentrations is accurate, sensitive, and easy to operate, meeting the requirements of TDM. It can serve as a foundation for further research on the pharmacokinetics of polymyxin and tigecycline, providing references for safe and effective clinical drug use.

## Conclusion

The HPLC–MS/MS method established in this study could simultaneously determine the plasma concentrations of colistin sulfate and tigecycline. The detection method was efficient, convenient, and accurate. It could quickly provide patients with plasma concentration data, adjust individualized medication schedules, and meet the requirements of further PK investigations of colistin sulfate and tigecycline. However, this study has limitations regarding the number of case and blood collection points; therefore, expanding the sample size for further PK/PD investigations is necessary.

## Data Availability

All data generated or analysed during this study are included in this article. Further enquiries can be directed to the corresponding author.
